# Removal of cadmium (II) from simulated wastewater by ion flotation technique

**DOI:** 10.1186/1735-2746-10-16

**Published:** 2013-02-07

**Authors:** Mohammad Hossein Salmani, Mojtaba Davoodi, Mohammad Hassan Ehrampoush, Mohammad Taghi Ghaneian, Mohammad Hossein Fallahzadah

**Affiliations:** 1 Department of Environmental Health Engineering, Faculty of Public Health, Shahid Sadoughi University of Medical Sciences, Yazd, Iran; 2 Department of Environmental Health Engineering, Faculty of Public Health, Tehran University of Medical Sciences, Tehran, Iran

**Keywords:** Cadmium, Removal, Ion flotation, Simulated wastewater

## Abstract

A separation technique which has recently received a sharp increase in research activities is “ion flotation”. This technique has four important advantages for treating wastewaters: low energy consumption, small space requirements, small volume of sludge and acting selectively. The present study aims to optimize parameters of ion flotation for cadmium removal in simulated wastewater at laboratory scale. It was obtained on the reaction between Cd^2+^ and sodium dodecylesulfate (SDS) collector followed by flotation with ethanol as frother. Test solution was prepared by combining the required amount of cadmium ion, SDS and necessary frother or sodium sulfate solution. All experiments were carried out in a flotation column at laboratory temperature (27°C), adjusted pH = 4 and 120 minutes. The different parameters (namely: flow rate, cadmium, SDS and frother concentrations and ionic strength) influencing the flotation process were examined. The best removal efficiency obtained at a collector-metal ratio of 3:1 in 60 min with flow rate of 150 mL/min was 84%. The maximum cadmium removal was 92.1% where ethanol was introduced at a concentration 0.4% to flotation column with above conditions. The obtained results were promising, as both cadmium and collector were effectively removed from wastewater. Hence, the application of ion flotation for metal ions removal from effluents seems to be efficient.

## Introduction

Heavy metals have been used by humans for thousands of years. Several adverse health effects have been known for a long time exposure to heavy metals and is even increased recently in some parts of the world, in particular in less developed countries [1]. Cadmium is a toxic metal occurring naturally in the environment and is considered as a pollutant emanating from industrial and agricultural sources. Exposure of human population to cadmium from air, food and water may produce effects in organs such as kidney, liver, lung, and cardiovascular, immune and reproductive systems [2]. Cadmium is efficiently retained in the kidney (half-time of 10–30 years) and the concentration is proportional to that in urine [3]. Recent data indicate that adverse health effects of cadmium exposure may occur at lower exposure level than previously anticipated [4,5]. Therefore, cadmium removal from water resources and industrial wastewater is necessary.

Numerous techniques such as chemical reduction, electrochemical treatment, ion exchange, precipitation and adsorption gas bobble separation have been reported for the separation, removal, and control of heavy metals [6,7]. Adsorptive bubble separation technique is becoming increasingly important for removing a wide variety of substances from wastewaters [8]. There are several separation techniques employing adsorption on gas bubbles; these methods are divided into two categories, foam separation and non foaming adsorptive bubble separation techniques. Foam separation techniques can be subdivided into foam fractionation and flotation. Flotation methods include ore flotation, macro flotation, colloidal flotation, ion flotation and precipitate flotation. Precipitate and adsorbing colloid flotation are processes that, together with ion flotation, possess some advantages for treating large volumes of wastewater solutions. Precipitate flotation requires precipitation of the metal species in preparation for subsequent flotation [9]. Adsorbing colloid flotation involves removal of metal ions by adsorption onto carrier floe such as Fe(OH)_3_ and Al(OH)_3_.

Ion flotation as an adsorptive separation technique has recently received a sharp increase in research activities. This process is a simple and effective method for separation and removal of metallic ions from wastewaters. Some works have been carried on for ion flotation of copper [10] and lead [11] from wastewater. Ion flotation may have several advantages for treating wastewater: low energy requirements, low residual concentration of metals, rapid operation, small space requirements, flexibility in applying the method to a variety of metals at various levels, and production of small volume of sludge [12]. This process can be influenced by many physical and chemical factors such as: type and concentration of collector and frother, time of flotation, ionic strength and ion metal. Since these factors are interrelated, ion flotation control is quite difficult to find the optimum condition [13]. It is believed that this process will be soon incorporated as a clean technology to treat water and wastewater [14].

In this study, the experiments were made to find the best conditions based on the maximum removal of ionic cadmium in a simulated wastewater for industrial application. The paper optimizes selective parameters for cadmium removal from simulated wastewaters by ion flotation technique.

## Materials and Methods

### Chemical reagent

All chemicals in this study were of analytical reagent grade from Merck Co. Sodium dodecyl sulfate (SDS), C_2_H_5_OH and CdSO_4_.8H_2_O were used as the surfactant, frother and the source of cadmium ion, respectively. A stock of Cd^2+^ solution of 1000 mg/L concentration was prepared by dissolving a predetermined amount of CdSO_4_.8H_2_O in distilled water. Appropriate standard solutions of Cd^2+^ were prepared in 1% HNO_3_ immediately before analysis by serial dilution of the stock solution. The ion strength was adjusted by Na_2_SO_4_.

### Apparatus

A Varian atomic absorption spectrophotometer (model AA-20) was used for determining concentration of Cd^2+^ at 228.8 nm. The pH of the solution was measured by a Jenway model 3320 pH meter. Flotation was carried out in a plexy glass column of 90 Cm height and 4 Cm in diameter. The column contained a stone-filter (10-15 μm, porosity of 4) at the end point for producing small air bubbles. An air compressor was used to generate air bubbles at controlled pressure of 1.1 atm. This compressor included a manometer for control of air flow rate.

### Methodology

Initial concentration contained 25 mg/L Cd^2+^. Each test solution was prepared by combining the required amount of cadmium salt stock solution, SDS stock solution and the necessary frother or sodium sulfate stock solution with distilled water to make up 0.5 liter of solution volume, filling approximately half of the column. The solution was stirred for approximately 15 min to ensure consistent mixing of all reagents and then pH of the solution was adjusted at 4 by H_2_SO_4_. Bubble generation began just after the solution was introduced into the column for 120 min. Samples were selected in bulk solution at 5, 15, 30, 60, 90 and 120 minutes while gas bubbles were producing. To assess the effect of parameters, several series of experiments were planed by variation of a: flow rate (50, 100 and 150 mL/min); b: SDS-Cd ratio (2:1, 2.5:1 and 3:1) and c: addition of ethanol (0.0, 0.4 and 0.8%). All samples and standard solutions for atomic absorption analysis were acidified in plastic bottles to a final pH of 1. Cd^2+^ concentration in the samples was determined in the flame mode, as descried in Varian analytical method [15,16]. All experiments were carried out at laboratory temperature (27±1°C). The removal percent of Cd^2+^was determined from the relationship:

$${R=C}_{i}-C_{f}/C_{i}\times 100$$

Where C_i_ and C_f_ denote the initial and final concentrations of Cd^2+^.

## Results

According to our previous knowledge of flotation processes and the fact that many physical and chemical factors affect ion flotation, in the present study, effects of air flow rate, concentration of collector and frother, and ionic strength were examined.

### Air flow rate effect

**Figure 1:**
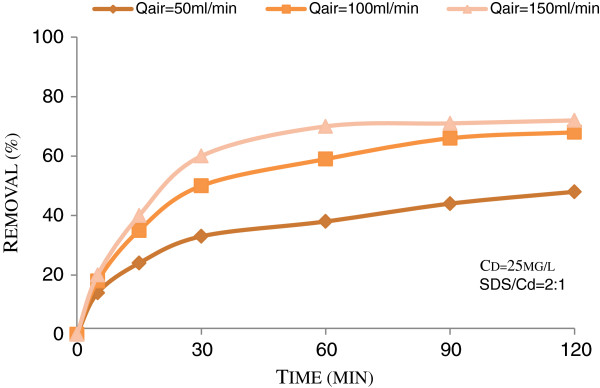
Effect of air flow rate on the floatability of cadmium in pH = 4 and T = 27°C.

Figure 1 shows the effect of air flow rate on cadmium flotation in presence of collector (metal ion of 2:1 molar ratio). As it can be seen, an increase in air flow rate increased cadmium removal. The maximum removal was observed at air flow rate of 150 mL/min, as 72%. The figure also shows that after 60 min of flotation, there are insignificant increases in flotation efficiency of cadmium as flotation time increases.

### SDS effect

**Figure 2:**
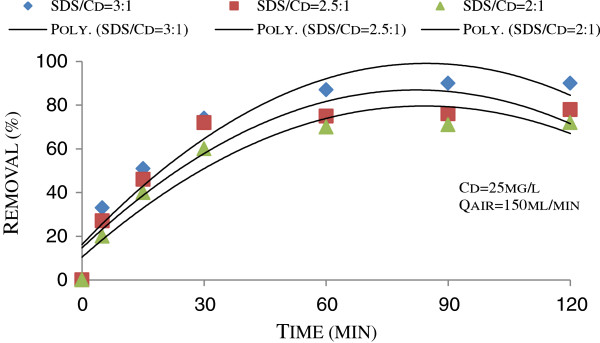
Effect of SDS concentration on the floatability of cadmium in pH = 4 and T = 27°C.

The effect of SDS concentration, acting as collector, on cadmium removal at air flow rate of 150 mL/min is shown in Figure 2. According to the figure, an increase in concentration of SDS resulted in an increase in cadmium removal. The best removal efficiency occurred at SDS: Cd molar ratio of 3:1, as 90%.

### Frother effect

**Figure 3:**
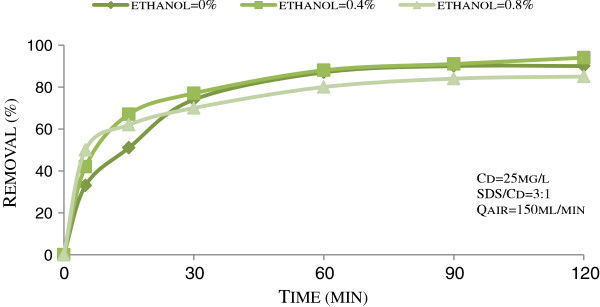
Effect of ethanol concentration on the floatability of cadmium in pH = 4 and T = 27°C.

Figure 3 presents cadmium flotation in presence of air flow rate of 150 mL/min and various concentrations of ethanol, as a frother. It can be seen that after 5 min flotation, the highest removal (50%) was observed at the highest concentration of ethanol (0.8% v/v). However, after 120 min, the highest removal was related to a concentration of 0.4% v/v of ethanol, as 94%.

### Ionic strength effect

**Figure 4:**
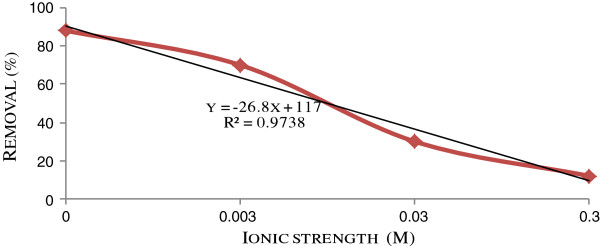
Effect of ionic strength on the floatability of cadmium in pH = 4 and T = 27°C.

In order to study the effect of ionic strength on ion flotation of cadmium, Na_2_SO_4_ at concentration of 10^-3^, 10^-2^ and 10^-1^ M was added to increase the ionic strength as 3×10^-3^, 3×10^-2^ and 3×10^-1^ M, respectively. The results obtained at 150 mL/min and 60 min flotation time are shown in Figure 4. According to the figure, increase in ionic strength adversely affected removal efficiency, so that with 0.3 M increase in ionic strength the cadmium removal dropped to 12%.

## Discussion

Flotation is a separation process and its main advantages are: high efficiencies (R>95%), rapidity, the possibility of removal and recovery of organic and inorganic species. The principle of floatability or non-floatability of chemical species is hydrophobicity. Substances are rendered hydrophobic by addition of appropriate collector, in which the polar groups are eliminated by adsorption living non-polar groups exposed to solution. Floatability is affected by various physical and chemical parameters such as pH, flotation time, and air flow rate, collector to metal ratio, frother concentration and ionic strength. The effect of pH on removal of cadmium by ion flotation was investigated along with literature search. Dissolved cadmium cations are known to precipitate out from the aqueous solution in pH of about 7, depending on the specific experimental conditions. It was found that for pH<6.0 cadmium cations are the soluble species. Precipitation starts at pH of about 8.0 and at pH= 9.0 all dissolved cadmium precipitates as insoluble cadmium hydroxide [17]. Hence, the experiments in this study were performed at pH =4.

Obtained results in all conditions for the removal of Cd^2+^ from wastewater (see Figures 1, 2 and 3) indicated that as flotation time increased, Cd removal increased. It also demonstrated that at the experimental conditions, the optimum removal of Cd occurred at 60 min flotation time. These results are in accordance with findings of Sreenivasarao and Scorzelli [18,19]. Based on the results that are shown in Figure 1, with three times increase at air flow rate, Cd removal was increased from 48% at 50 mL/min to 72% at 150 mL/min. This result confirms other investigations [11] and may be explained by increasing in air bubble production in the solution, resulted from air flow rate increases.

As can be seen from Figure 2, for 120 min flotation, 1.5 times increase in SDS concentration resulted in 1.25 times increase in Cd removal. Improved metal ion removal with an increase in collector-metal ratio has also been observed in other investigations [20,21]. In this case, increased number of SDS molecules compared to cadmium ions can be one of the reasons of improved Cd removal. The numbers of SDS molecules were 26.7×10^19^, 33.4×10^19^ and 40.1×10^19^ in case of SDS-Cd ratio of 2, 2.5 and 3, respectively. As a net result, adsorption of Cd^2+^ to DS^-^ stimulates as DS^-^ ions are increased in the solution. Another reason may relate to surface tension decrease resulted from SDS concentration increases, that stimulate rising air bubbles and floating Cd-DS species.

The effects of ethanol, as a frother, on the ion flotation of cadmium are presented in Figure 3. It can be seen that at initial flotation times, (5 min), Cd removal was directly related to the ethanol concentrations. While at longer flotation times, (120 min), the higher concentration of ethanol resulted in lower Cd removal. Overally, ethanol on an optimum range can improve the ion flotation of cadmium. Duyvesteyn found that alcohols, as frother, have a set of different effects on ion flotation process. Their positive effects are attaching to bubbles, resulting in finer and stranger bubbles. They can also stabilize metal-collector species and improve their floatability. However, alcohol based frother at high concentrations are deleterious for ion flotation, because of the competition between Cd-DS complexes and alcohol molecules for adsorption sites of air bubbles and also stabilized metal-collector complexes [22].

Figure 4 shows that the higher ionic strength, the lower cadmium removal will be achieved. This finding is according to other investigations [23,24]. In ion flotation, an interaction must occur between an ionic surfactant and oppositely charged metal ion to remove non surface active ions from aqueous solutions. In presence of SO_4_^2-^, cadmium in the form of Cd^2+^ are converted to the neutral and anionic complexes such as CdSO_4_, Cd(SO_4_)_2_^2-^ and Cd(SO_4_)_3_^4-^ with low potential to react with anionic surfactant, DS^-^. On the other hand, Na_2_SO_4_ in high concentrations resulted in a competition between Cd^2+^ residual and Na^+^ for DS^-^, resulting Cd removal to be lower than what theoretically expected [25].

## Conclusion

The main objective of this study was to optimize the parameters in cadmium ion flotation. To achieve the aim, effective parameters were chosen considering different experiments. Comparison of the aforementioned results showed that the optimum time for best removal efficiency of cadmium ion flotation was 60 min with flow rate of 150 mL/min. Also, it was shown that the removal efficiency of Cd^2+^ was 82.5% at the metal-collector ratio of 1:3 and pH = 4. The presence of frother at a concentration of 0.4% v/v increased removal and the maximum removal was obtained 94%.

The removal of cadmium ions by ion flotation process is very sensitive to the ionic strength. So the ionic strength of wastewaters must be decreased by an acid (such as H_2_SO_4_) before flotation. Subsequently, the application of ion flotation for cadmium ions removal from aqueous dilute solutions seems to be efficient.

## Competing interests

The authors declare that they have no competing interests.

## Authors’ contribution

MHS and MD participated in the design of the study and draft the manuscript. MD carried out the experimental studies. MHE helped to design and draft the manuscript. MHF performed statistical analysis of the collected data. MTG read the manuscript. All authors read and approved the final manuscript.
